# Evaluation of primary internal limiting membrane peeling in cases with rhegmatogenous retinal detachment

**DOI:** 10.1186/s40942-020-00213-4

**Published:** 2020-05-07

**Authors:** Mohamed Esmail Abdullah, Hossam Eldeen Mohammad Moharram, Ahmed Shawkat Abdelhalim, Khaled Mohamed Mourad, Mohamed Farouk Abdelkader

**Affiliations:** 1 Faculty of Medicine, Minia University, Minia, Egypt; 2 Department of Ophthalmology, Minia University Hospital, Minia, 61111 Egypt

**Keywords:** Retina, Vitreous body, Vitreoretinal surgery, Peeling of internal limiting membrane (ILM), Vitrectomy, Retinal detachment, Electroretinography, Optical coherence tomography

## Abstract

**Background:**

Epiretinal membranes (ERMs) have been reported after pars plana vitrectomy (PPV) for rhegmatogenous retinal detachment (RRD). Peeling of the internal limiting membrane (ILM) can prevent post-PPV ERM formation but has a potential negative impact on macular structure and function.

**Purpose:**

To investigate the anatomical and functional outcomes of ILM peeling during PPV for primary RRD.

**Methods:**

This was a prospective nonrandomized study that included 60 eyes of 60 patients with a primary macula-off RRD and less than grade C proliferative vitreoretinopathy (PVR). Eyes were allocated into 2 groups; Group A underwent PPV without ILM peeling and Group B had ILM peeling. At postoperative month 6, all patients underwent retinal imaging using spectral domain optical coherence tomography (OCT) and OCT angiography and macular function was assessed using multifocal electroretinogram (mfERG). Baseline characteristics and postoperative anatomical and visual outcomes were recorded and statistically analyzed.

**Results:**

We enrolled 30 eyes of 30 patients in each group. In Group A, mean age was 44.6 years, while the mean age of Group B patients was 49.9 years. Postoperative LogMAR visual acuity was significantly better in Group A than in Group B (p < 0.001). ERMs were demonstrated on OCT in 13.3% of Group A and none of Group B patients (p = 0.04). Retinal dimples were found in 53.3% of Group B and none of Group A eyes (p < 0.001). OCTA showed a greater vessel density of the superficial capillary plexus (SCP) in Group A compared to Group B eyes (p = 0.046), while no difference was found regarding deep capillary vessel density (p = 0.7). Mean amplitude of mfERG P1 wave was significantly higher in Group A eyes than in Group B (p = 0.002). Both the SCP vessel density and P1 amplitude were positively correlated with visual acuity (p < 0.001).

**Conclusion:**

This study suggests that ILM peeling prevents ERM development in eyes undergoing PPV for uncomplicated macula-off RRD, but potential damage to macular structure and function were found.

*Trial registration* Retrospectively registered on 09/24/2019 on ClinicalTrials.gov with an ID of NCT04139811.

## Background

Internal limiting membrane (ILM) peeling has become an integral step during pars plana vitrectomy (PPV) for managing different macular pathologies, such as macular holes, vitreomacular traction (VMT) syndrome and epiretinal membranes (ERM) [1]. Removal of the ILM has been shown to decrease the recurrence rate of idiopathic ERMs after their surgical removal [2].

ERMs may develop after successful vitrectomy for repair of rhegmatogenous retinal detachment (RRD) with an incidence that varies from 6 to 48% according to published literature [3]. Risk factors for post-PPV ERM development include multiple, large or posterior retinal breaks, as well as a longer duration of macular detachment [4, 5].

ERM development after RRD repair is mostly attributed to migration of retinal pigment epithelial (RPE) cells through retinal breaks and their proliferation on the macular surface. ILM peeling in the setting of primary PPV would remove the scaffold required for these proliferating cells [4]. Several studies evaluated the role of ILM peeling in preventing post-PPV ERM formation and its impact on macular microstructure and function [6–14]. The purpose of this study was to assess the anatomical and functional outcomes after ILM peeling during PPV for primary macula-off RRD utilizing optical coherence tomography (OCT), OCT angiography (OCTA) and multifocal electroretinogram (mfERG).

## Methods

This was a prospective comparative nonrandomized study. Patients were recruited and surgeries performed at the Department of Ophthalmology, Minia University, Minia, Egypt during the period from March 2017 to September 2019. A written informed consent was obtained from all study participants after thorough explanation of the risks and benefits of surgery and nature of the study. The study was approved by local Ethical Committee of Faculty of Medicine, Minia University and adhered to the tenets of the Declaration of Helsinki.

Patients who were older than 18 years and presented with a primary macula-off RRD with less than grade C proliferative vitreoretinopathy (PVR) were eligible for inclusion in the study. Eyes were excluded if they had prior vitreoretinal surgery or intravitreal injections, combined rhegmatogenous-tractional RD, other macular pathologies, history of glaucoma or corneal opacities that might hinder acquisition of good quality OCT and OCTA images.

Eyes were allocated into 2 groups; Group A underwent PPV without ILM peeling and Group B had ILM peeling. All eyes were operated within 1 week of presentation to avoid progression to advanced PVR. Eyes that had significant cataract underwent phacoemulsification and primary intraocular lens implantation prior to vitrectomy. All PPV surgeries were performed by a single experienced surgeon (MFA). A standard 3-port 23-gauge PPV, using valved one-step transconjunctival trocar and cannula system (DORC International, Zuidland, The Netherlands), was performed for all eyes and perfluorocarbon liquid (PFCL) was used to flatten the retina followed by peripheral vitreous shaving, Endolaser treatment around the causative break(s) and 360° endolaser applied to the periphery. Silicone 1000 cSt was used as a tamponade in all eyes. In Group B eyes, Brilliant Blue G dye 0.025% (ILM-BLUE; DORC International, Zuidland, The Netherlands) was instilled over the detached macula in a fluid-filled eye and left for 30 s followed by PFCL injection. An ILM edge was created at the lower temporal arcade using a diamond-dusted Tano scraper and peeling was completed using an ILM peeling forceps (DORC International). ILM peeling was extended to the major arcades.

Patients were seen at 1 day, 1 week, 1 month and 3 months postoperatively. Silicone oil removal was performed at the 3rd postoperative month, combined with phacoemulsification with intraocular lens (IOL) implantation for all phakic eyes.

Spectral domain (SD) OCT and OCTA imaging was performed for all eyes in the absence of silicone oil at the 6th postoperative month after the original surgery. OCT and OCTA imaging were done using Avanti RTVue-XR platform (Optovue Inc., Fremont, CA) which uses a split-spectrum amplitude-decorrelation angiography (SSADA) algorithm. Two consecutive B-scans are obtained at each location before moving to another one. Each OCTA volume contains 304 × 304 A-scans. In order to minimize motion artifacts, 2 orthogonal OCTA volumes are obtained. The Angio Retina protocol was used for OCTA image acquisition, with a scan area of 6 × 6 mm. Automated segmentation was used to define the different vascular plexuses. OCT parameters evaluated were the central macular thickness (CMT), presence or absence of ERM and retinal dimples. OCTA images were evaluated for vessel density (VD) of both the superficial capillary plexus (SCP) and deep capillary plexus (DCP). The vessel area density (VD) was calculated using the Vessel Density Map of the AngioAnalytics software (RTVue-XR version: 2017.1.0.151, Optovue) as the percentage area occupied by vessels relative to the total scan area at the level of the SCP and DCP. All OCT and OCTA images were evaluated by 2 experienced observers.

Multifocal electroretinogram (mfERG) was recorded for all eyes at the 6th postoperative month using RetiPort System (Roland Consult, Brandenburg, Germany). The test was performed according to the standards of the International Society of Clinical Electrophysiology of Vision (ISCEV) [15]. The P1 amplitude was evaluated.

In mfERG recording procedure, an active HK-loop electrode (Roland Consult) was applied to the lower conjunctival fornix after 10 min of light adaptation and pupil dilatation with tropicamide 1% eye drops. A ground electrode was applied to the forehead and a reference electrode was connected to the ipsilateral temple. The standard mfERG visual stimulus was used, which consists from 61 hexagons covering the central 25–30° of the visual field, and presented on a 20-inch monitor at a distance of 33 cm. For each hexagon, the amplitude of P1 (defined as the difference between the trough of N1; first negative wave, and the peak of P1; second positive wave) was calculated.

### Statistical analyses

The collected data were coded, tabulated and statistically analyzed using IBM SPSS Statistics for Windows, Version 25.0 (IBM Corp., Armonk, NY). Parametric quantitative data were expressed as mean ± standard deviation, non-parametric quantitative data as median and interquartile range (IQR), and categorical data as numbers and percentages. Distribution of data was done using Kolmogorov–Smirnov test. Comparison between both study groups was done using Independent Samples T test for parametric quantitative data and Mann–Whitney test for non-parametric quantitative data. Analyses were done for qualitative data using Chi square test (if < 20% of cells had an expected count < 5) and Fisher’s exact test (if > 20% of cells had an expected count < 5). p value < 0.05 was considered significant. Correlation analysis between variables was done applying Spearman’s ranked correlation test for non-parametric data.

## Results

### Baseline characteristics

We enrolled 30 eyes of 30 patients in each group. In Group A, mean age was 44.6 years and 24 patients were males (80%). The mean age of Group B participants was 49.9 years and males constituted 76.7% (23 patients). Both groups were balanced regarding their age, sex, lens status, extent of RD and number of breaks (Table 1).

**Table 1:** Baseline characteristics of both study groups

Characteristic		Group A	Group B	p value
		N = 30	N = 30	
Age	Range	(20–60)	(20–73)	0.095
	Mean ± SD	44.6 ± 11.2	49.9 ± 13.1	
Sex				
Male	No. (%)	24 (80%)	23 (76.7%)	0.754
Female	No. (%)	6 (20%)	7 (23.3%)	
Extent of detachment				
Subtotal	No. (%)	19 (63.3%)	22 (73.3%)	0.405
Total	No. (%)	11 (36.7%)	8 (26.7%)	
Lens status				
Phakic	No. (%)	10 (33.3%)	11 (36.7%)	0.314
Cataract	No. (%)	3 (10%)	0 (0%)	
Pseudophakic	No. (%)	17 (56.7%)	19 (63.3%)	
No. of breaks				
Single	No. (%)	20 (66.7%)	21 (70%)	0.781
Multiple	No. (%)	10 (33.3%)	9 (30%)	
Best-corrected LogMAR VA	Mean ± SD	1.9 ± 0.5	1.9 ± 0.3	0.572

*LogMAR* logarithm of minimum angle resolvable, *VA* visual acuity

### Visual outcomes

All eyes in both groups had macula-off RRD, defined as complete involvement of the fovea by subretinal fluid as detected clinically by fundus biomicroscopy, with a mean preoperative LogMAR BCVA of 1.9 ± 0.5 in group A and 1.9 ± 0.3 in group B. Postoperative LogMAR BCVA was significantly better in Group A (non-ILM peeling) than in Group B at the 6th postoperative month (p < 0.001). Mean LogMAR BCVA was 0.6 ± 0.2 in Group A (range 0.3–0.9) and 0.9 ± 0.15 in Group B (range 0.6–1.0).

### OCT parameters

**Fig. 1:**
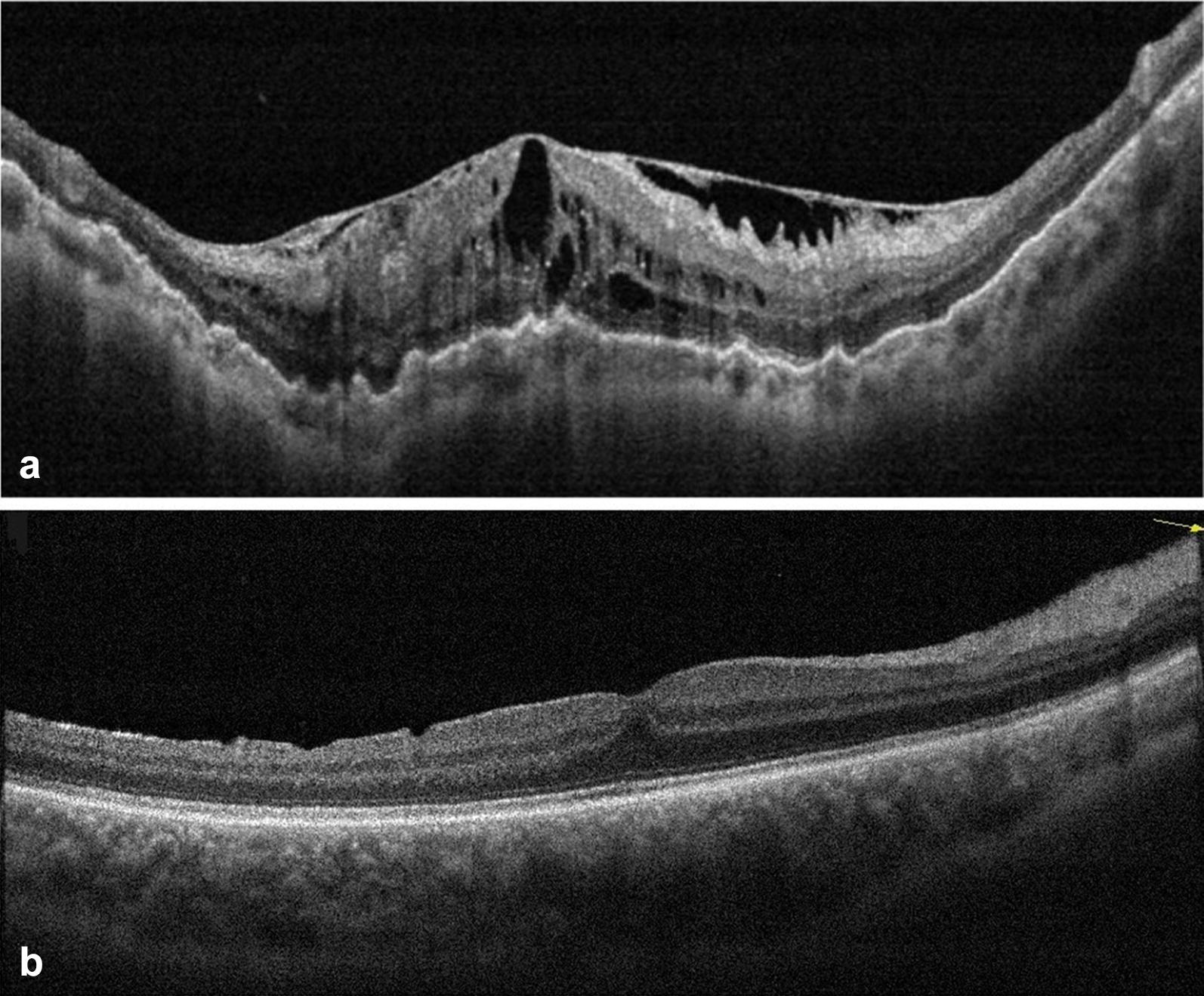
**a** B-scan SD-OCT of the macula of a Group A patient showing a thick epiretinal membrane (ERM) causing macular pucker, thickening and cystoid degeneration after pars plana vitrectomy. **b** B-scan SD-OCT of the macula of a Group B patient showing dimples on the inner retinal surface after pars plana vitrectomy and peeling of the internal limiting membrane (ILM)

ERMs were demonstrated on SD OCT in 4 cases (13.3%) of Group A and none of Group B patients at 6 months (p = 0.04). Only 3 eyes in Group A had significant cataract and underwent phacovitrectomy, and none of them developed an ERM. No eyes in Group B had significant cataract that required lens removal before PPV. Retinal dimples were evident in 16 Group B patients (53.3%) and none of Group A patients (p < 0.001). No significant difference was found between both groups regarding postoperative CMT (262.1 ± 54.4 μm in Group A vs 262.3 ± 38.7 μm in Group B, p = 0.99) (Fig. 1).

### Octa parameters

**Fig. 2:**
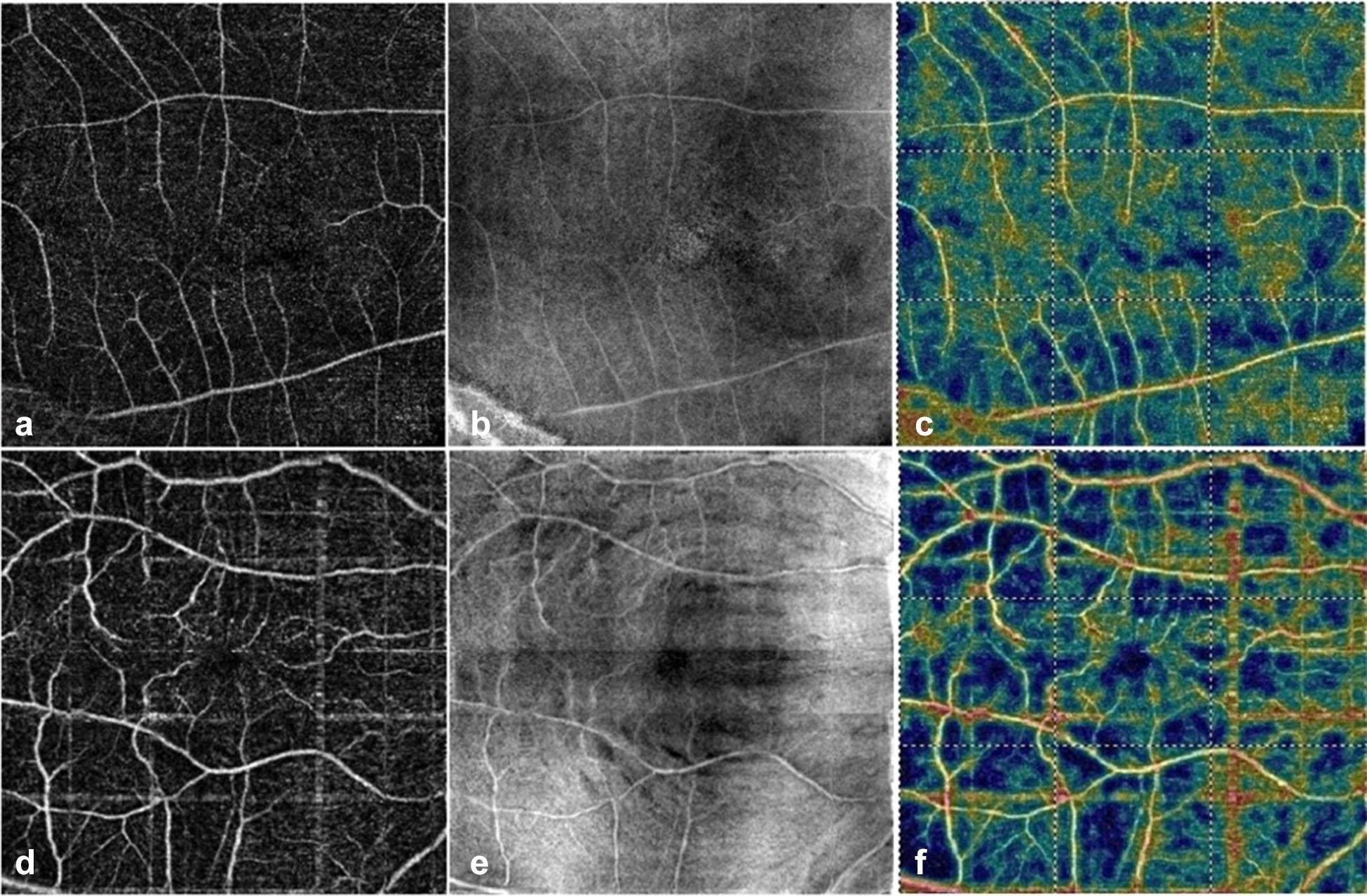
Top row represents a Group A eye. **a** An optical coherence tomography angiography (OCTA) macula 6 × 6 mm scan showing the slab representing the superficial capillary plexus (SCP). **b** En-face OCT image that corresponds to the OCTA slab in A. **c** The color-coded flow density map of the superficial vessel density (warmer colors represent greater flow density). Bottom row represents a Group B eye. **d** An OCTA macula 6 × 6 mm scan showing the slab representing the superficial capillary plexus (SCP). **e** En-face OCT image that corresponds to the OCTA slab in D showing retinal dimples at the level of the nerve fiber layer. **f** The color-coded flow density map of the superficial vessel density

Mean vessel density of the SCP was 44.8 ± 6.4% in Group A and 41.7 ± 5.5% in Group B, a difference that barely reached statistical significance (p = 0.046). Moreover, greater SCP vessel density was significantly correlated with better visual acuity (r = − 0.83, p < 0.001). No significant difference in VD of the DCP was found between both groups (42.3 ± 5.8% in Group A vs 41.7 ± 5.4% in Group B, p = 0.7) (Fig. 2).

### Multifocal ERG

**Fig. 3:**
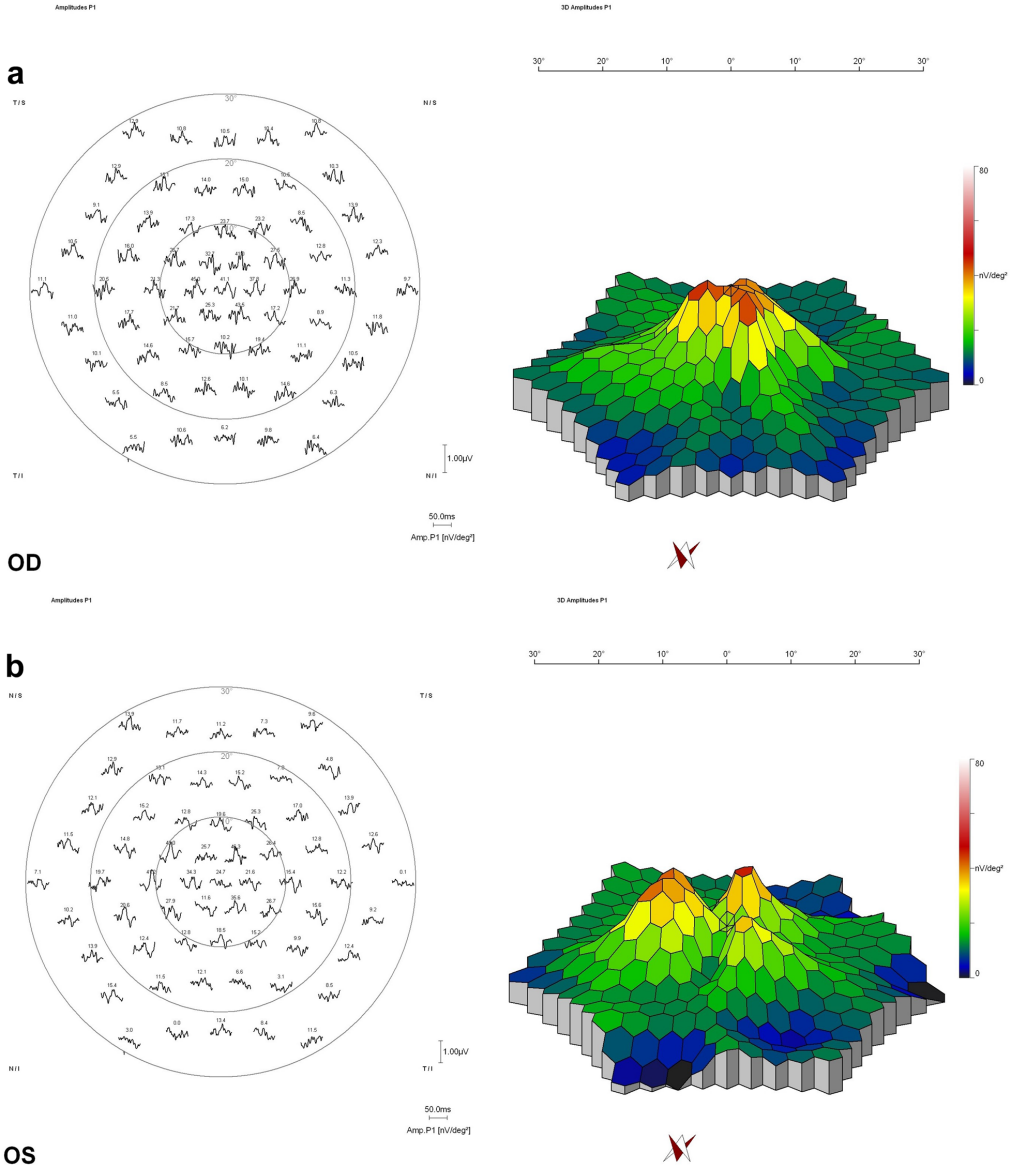
**a** Multifocal electroretinogram (mfERG) of a Group A eye. Left. Amplitude of P1 in nV/deg^2^ in topographic display around the fovea. Right. Three-dimensional topography of P1 amplitude (nV/deg^2^). **b** Multifocal electroretinogram (mfERG) of a Group B eye. Left. Amplitude of P1 in nV/deg^2^ in topographic display around the fovea. Right. Three-dimensional topography of P1 amplitude (nV/deg^2^)

Mean amplitude of P1 wave was significantly higher in Group A eyes (40.1 ± 9.8 nV/deg^2^) compared to Group B (30.9 ± 11.8 nV/deg^2^, p = 0.002). Furthermore, the P1 amplitude was strongly correlated to BCVA (r = -0.9, p < 0.001) (Fig. 3). Table 2 summarizes the postoperative outcomes for both groups.

**Table 2:** Post-operative outcomes in both groups at 6 months

Outcome	Group A	Group B	p value
	N = 30	N = 30	
LogMAR BCVA			
Range	(0.3–0.9)	(0.6–1)	< 0.001*
Mean ± SD	0.6 ± 0.2	0.9 ± 0.15	
CMT			
Range	(162–350)	(209–334)	0.987
Mean ± SD	262.1 ± 54.4	262.3 ± 38.7	
VD-SCP			
Range	(35.29–51.53)	(30.11–52)	0.046*
Mean ± SD	44.8 ± 6.4	41.7 ± 5.5	
VD-DCP			
Range	(31.5–53.1)	(30.1–53.1)	0.674
Mean ± SD	42.3 ± 5.8	41.7 ± 5.4	
ERM			
No. (%)	4 (13.3%)	0 (0%)	0.04*
Dimples			
No. (%)	0 (0%)	16 (53.3%)	< 0.001*
P1 amplitude			
Mean ± SD	40.1 ± 9.8	30.9 ± 11.8	0.002*

*BCVA* best-corrected visual acuity, *CMT* central macular thickness, *VD-SCP* vessel density of superficial capillary plexus, *VD-DCP* vessel density of deep capillary plexus, *ERM* epiretinal membrane

* Italic type indicates statistical significance

## Discussion

ILM peeling has been shown by several investigators to prevent ERM formation after vitrectomy for repair of RRD of different degrees of complexity [6–14]. We included only eyes with primary RRD and no more than grade B PVR, because we sought to assess the benefit of ILM peeling as a routine step during repair of uncomplicated RRD. Advanced PVR could be associated with higher risk for postoperative ERM development and inclusion of these eyes might have confounded our anatomical results [13, 16]. We also excluded eyes with fovea-sparing RRDs to avoid any potential complications of performing ILM peeling on a non-diseased macula.

A recent meta-analysis of studies comparing ILM peeling versus no peeling during PPV for RRD found the incidence of postoperative macular ERM formation to be significantly lower in patients who had ILM peeling, but this bared no significant effect on postoperative visual outcomes [3].

In agreement with previous studies, eyes that had ILM peeling in our study developed no ERMs at postoperative month 6, while an ERM was demonstrated on SD OCT in 4 eyes (13.3%) that had no ILM peeling (p = 0.04). Indeed, the incidence of postoperative ERM development in eyes that underwent ILM peeling during PPV for RRD ranged from 0 to 9% in several publications [6, 7, 9, 13, 14]. This is to be expected as ILM peeling ensures complete removal of the posterior hyaloid cortex and deprives RPE cells from the support they need to proliferate on the macular surface [17, 18].

We performed a thorough vitreous base shaving in all eyes and peripheral 360 degrees endolaser was done in order to avoid missing any minute holes, which might not have been visualized during surgery, which may lead to RD recurrence. Although peripheral 360 degrees endolaser was performed in all eyes in both groups, none of the eyes in group B developed an ERM while ERMs were demonstrated in 4 eyes (13.3%) of Group A, suggesting that peripheral endolaser did not appear to increase incidence of ERMs.

We used silicone 1000 cSt in all eyes due to the frequent shortages of expansile gases in our locality, while silicone oil is always available, to ensure that all eyes received the same tamponade and eliminate any confounding variable that might affect postoperative macular anatomy and function.

Several studies have shown that ILM peeling may cause microstructural mechanical damage to the retina in the form of retinal dimples, dissociated nerve fiber layer (DONFL) and focal retinal thinning [19]. This trauma has been attributed to the impact on the macular surface of instruments used to peel the ILM [20, 21].

Retinal dimples developed postoperatively in 16 Group B eyes (53.3%) and none of Group A eyes (p < 0.001). Recent studies demonstrated inner retinal dimples in 100% of eyes that underwent PPV with ILM peeling for RRD repair [9, 22]. The incidence of retinal dimples was reported to be 68% after ILM removal for full-thickness and lamellar macular holes [23]. Retinal dimples are hypothesized to be the result of diffuse damage to Müller cell end-feet. The impact of these changes on macular function is still unclear [24, 25]. Concordant with the results of Eissa and associates [9], we found no significant difference in postoperative CMT between the 2 groups.

To the best of our knowledge, this is the first study to use OCTA for evaluating the effect of ILM peeling on macular microvasculature after PPV for RRD. OCTA is a recent addition to the expanding arsenal of retinal imaging modalities, and has provided novel insights into the pathogenesis of different macular diseases [26]. Mastropasqua and colleagues used OCTA to study SCP changes after ILM peeling for idiopathic ERMs. They noted a reduction in the SCP vessel density which was evident in areas of microstructural alterations on SD OCT such as swelling of the arcuate nerve fiber layer. They hypothesized that these changes resulted from the direct surgical trauma to the inner retina which contains the SCP [27]. Similarly we found a marginally significant reduction in SCP vessel density among Group B eyes compared to Group A (p = 0.046). However, no significant difference existed between both groups regarding the DCP vessel density, most likely because of the DCP was not directly impacted by surgical manipulation.

Group A eyes had significantly better BCVA than Group B eyes at final follow-up (p < 0.001). Furthermore, postoperative vision significantly and positively correlated with SCP vessel density and mfERG P1 wave amplitude (p < 0.001). This comes in agreement with Eissa et al. [9] Other studies have reported a trend towards better visual acuity in non-ILM peeling eyes compared to eyes that had ILM peeling, though this difference did not reach statistical significance [8, 10, 14]. Interestingly, Aras et al. demonstrated similar visual outcomes between eyes that had ILM peeling during PPV for complex RRDs compared to those with no ILM peeling [12]. This might indicate that the magnitude of effect that ILM peeling had on visual function was less meaningful in eyes with complicated pathology and, hence, ILM peeling could be more justified in such context.

Multifocal ERG is an objective method to evaluate macular function. The P1 peak is thought to originate from the inner retinal cells, namely bipolar and Muller cells and this is why we chose to use P1 amplitude in our analysis [15, 28]. We found a significant reduction in P1 amplitude in Group B eyes compared to Group A and this reduction correlated with lower BCVA and SCP vessel density (p < 0.001).

Other studies utilized mfERG to assess macular function after ILM peeling for idiopathic ERMs. Lim et al. found nonsignificant reduction in postoperative P1 amplitude compared to preoperative values. This reduction did not recover at 1 year postoperatively, despite visual and anatomical improvement [29]. Another study found slight reduction in mfERG amplitudes that was accompanied by an asymptomatic decrease in visual field sensitivity in a small group of patients after ILM peeling for idiopathic macular pucker [30].

Eissa et al. found significant reduction in mean and foveal retinal sensitivity as measured by microperimetry in the ILM versus none-ILM peeling group. Both microperimetry measures correlated with final BCVA [9]. All these findings point the functional deficit that can be caused by ILM peeling even in asymptomatic patients.

## Conclusions

In conclusion, this study suggests that ILM peeling prevents ERM development in eyes undergoing PPV for uncomplicated macula-off RRD, but potential damage might occur to macular structure and function. Limitations of our study include the small sample size, lack of randomization and relatively short follow-up period. Among the strengths of our study are the use of strict selection criteria and utilization of recent modalities to assess macular structure and function such as OCTA and mfERG. It is our belief that ILM peeling should be reserved for more complex cases of RD with advanced PVR rather than be incorporated as a routine step during primary RD repair.

## Data Availability

The datasets used and/or analysed during the current study are available from the corresponding author on reasonable request.

## Abbreviations

BCVA: Best-corrected visual acuity
CMT: Central macular thickness
DCP: Deep capillary plexus
DONFL: Dissociated optic nerve fiber layer
ERM: Epiretinal membrane
ILM: Internal limiting membrane
IOL: Intraocular lens
IQR: Interquartile range
ISCEV: International Society of Clinical Electrophysiology of Vision
LogMAR: Logarithm of minimum angle resolvable
mfERG: Multifocal electroretinogram
OCTA: Optical coherence tomography angiography
PFCL: Perfluorocarbon liquid
PPV: Pars plana vitrectomy
PVR: Proliferative vitreoretinopathy
RPE: Retinal pigment epithelium
RRD: Rhegmatogenous retinal detachment
SCP: Superficial capillary plexus
SD OCT: Spectral domain optical coherence tomography
SSADA: Split-spectrum amplitude-decorrelation angiography
VMT: Vitreomacular traction
